# Supplementary material for: The discovery of novel noncoding RNAs in 50 bacterial genomes
Source: Nucleic Acids Res. 2024 Apr 22;52(9):5152–65. doi: 10.1093/nar/gkae248 (PMC11109978; doi:10.1093/nar/gkae248)
Supplement: gkae248_Supplemental_Files [file gkae248_supplemental_files.zip › Narunsky 50 Genomes RNA Motifs Files.zip!/Publication/68-C_sticklandii/Figures/LRC-68-5-NC_014614_1_IGR_17_2644058_2644503.sto.pdf]

LRC-68-5-NC_014614_1_IGR_17_2644058_2644503.sto.r2r

5′-○○RAG●YA●AG●AU●●●●CR●A●AA●UA●CYACARC●●●ACAAACGU●UCARG●UGCRUAUAAU●AAYRUUU●●●U○○○AA●●AAG●UG●RAG●●●U●AAGCUAA●AG●●●UGUGGAAG○○UCY●AAC●RC●●CYARCAG●●RA○○○Y●YUY●U●●UUUU●●UCUUAYA●UR●RUY●○○○○○YUUA●C●R○○○○○○○○○○○○●UGA●●●●G●RCA●UU●GYYAA●R●●UUG●GUAUC●A●UAU●AYA●AAYR●A○○○●RYAGY●UUYUUYGAUGGU●UY●○○○○○○○○○○○○○○○○○
